# *AtPV42a* and *AtPV42b* Redundantly Regulate Reproductive Development in *Arabidopsis thaliana*

**DOI:** 10.1371/journal.pone.0019033

**Published:** 2011-04-20

**Authors:** Lei Fang, Xingliang Hou, Li Yen Candy Lee, Lu Liu, Xiaojing Yan, Hao Yu

**Affiliations:** Department of Biological Sciences and Temasek Life Sciences Laboratory, National University of Singapore, Singapore, Republic of Singapore; Ecole Normale Superieure, France

## Abstract

**Background:**

The conserved SNF1/AMPK/SnRK1 complexes are global regulators of metabolic responses in eukaryotes and play a key role in the control of energy balance. Although α-type subunits of the SnRK1 complex have been characterized in several plant species, the biological function of β-type and γ-type subunits remains largely unknown. Here, we characterized *AtPV42a* and *AtPV42b*, the two homologous genes in *Arabidopsis*, which encode cystathionine-β-synthase (CBS) domain-containing proteins that belong to the PV42 class of γ-type subunits of the plant SnRK1 complexes.

**Methodology/Principal Findings:**

Real-time polymerase chain reaction was performed to examine the expression of *AtPV42a* and *AtPV42b* in various tissues. Transgenic plants that expressed artificial microRNAs targeting these two genes were created. Reproductive organ development and fertilization in these plants were examined by various approaches, including histological analysis, scanning electron microscopy, transmission electron microscopy, and phenotypic analyses of reciprocal crosses between wild-type and transgenic plants. We found that *AtPV42a* and *AtPV42b* were expressed in various tissues during different developmental stages. Transgenic plants where *AtPV42a* and *AtPV42b* were simultaneously silenced developed shorter siliques and reduced seed sets. Such low fertility phenotype resulted from deregulation of late stamen development and impairment of pollen tube attraction conferred by the female gametophyte.

**Conclusions:**

Our results demonstrate that *AtPV42a* and *AtPV42b* play redundant roles in regulating male gametogenesis and pollen tube guidance, indicating that the *Arabidopsis* SnRK1 complexes might be involved in the control of reproductive development.

## Introduction

As sessile organisms, plants are exposed to a constantly changing environment. It is therefore essential for them to sense and integrate endogenous and environmental stimuli to generate suitable cell responses for optimizing growth and development [1]. The control of energy balance is one of the crucial factors for such adaptive processes in plants, which involves a group of plant protein kinases, the SNF1-Related Kinase 1 (SnRK1) family [2].

SnRK1 is a serine/threonine kinase that has a catalytic domain similar to that of Sucrose non-fermenting 1 (SNF1) from yeast and AMP-activated protein kinase (AMPK) from mammals [2], [3]. In yeast, SNF1 is one of the main regulators of carbon metabolism and mediates the diauxic shift from fermentative to oxidative metabolism in response to glucose starvation [4], [5]. AMPK, the mammalian counterpart of SNF1, is an energy sensor that regulates energy balance by activating the processes that produce energy, while inhibiting those that consume energy [6]–[8]. In plants, SnRK1-type kinases play an important role in the global regulation of metabolism, and are also involved in plant development and stress responses [1]. SnRK1s from different plant species can complement the yeast *snf1△* mutant phenotype, demonstrating an evolutionary conservation in their function [9]–[15].

SNF1, AMPK-α, and SnRK1 serve as the catalytic α-subunits that are associated with other two regulatory subunits (β-type and γ-type) in the conserved heterotrimeric kinase SNF1/AMPK/SnRK1 complexes found in fungi, mammals and plants [1]. Association of the three subunits in SNF1/AMPK/SnRK1 complexes is differentially regulated by various hormonal and environmental signals, cell and tissue types, and developmental stages. In yeast, β subunits anchor α and γ subunits, thus directing the kinase complexes into their targets or specific subcellular localizations, while γ subunits function in activating the kinase activity of α subunits [16]–[19].

Three γ-type subunits (AMPKγ1, AMPKγ2, AMPKγ3) in mammals have been identified as being homologous to the γ subunit of the SNF1 complex, SNF4, in yeast [20]. Furthermore, phylogenetic analysis of SNF4-like plant proteins has revealed three subgroups of γ-type subunits in plants: AKINβγ-, AKINγ-, and PV42-type proteins [3], [15], [21]–[23]. While it has been shown that AKINβγ contributes to SnRK1 heterotrimeric complexes in *Arabidopsis* and is possibly involved in plant-pathogen interactions [23], the biological function of AKINγ- and PV42-type proteins remains unclear.

The conservation among γ-type subunits in fungi, mammals and plants partly lies in the four cystathionine-β-synthase (CBS) domains found in these proteins [1], [24]. The CBS domain was first discovered in the genome of the archaebacterium *Methanococcus jannaschii* [24], [25]. It is about 60 residues long, and composed of a sheet of three β strands packed with two α helices. CBS domains have been found to bind to metallic ions such as Mg^2+^ and adenosyl compounds such as AMP, ATP, and S-adenosyl-L-methionine, which may trigger a conformational change in the CBS domains, thus regulating the activity of associated enzymatic domains [26]–[28]. The CBS domain-containing proteins comprise a large family of evolutionarily conserved proteins that have been found in all kingdoms of life, among which the mammalian ones are so far the best characterized. In humans, CBS domain-containing proteins are highly diversified and have been found to undertake various biological roles, ranging from metabolic enzymes and transcriptional regulators to ion channels and transporters [29]. In contrast, very few information is available for the CBS domain-containing proteins in plants. So far, 48 *Arabidopsis* proteins have been designated as CBS domain-containing proteins [29], which include γ-type subunits of the SnRK1 complex.

In this study, we show that *AtPV42a* and *AtPV42b*, the two homologous genes in *Arabidopsis*, encode CBS domain-containing proteins that belong to the PV42 class of γ-type subunits of the plant SnRK1 complexes. They are expressed in different tissues throughout the developmental stages of *Arabidopsis*. Artificial microRNA-mediated silencing of both *AtPV42a* and *AtPV42b* exhibits the defects in late stamen development and pollen tube attraction conferred by the female gametophyte, which results in reduced seed sets. These results suggest that *AtPV42a* and *AtPV42b* play redundant roles in regulating male gametogenesis and pollen tube guidance in *Arabidopsis*.

## Results

### AtPV42a and AtPV42b are putative γ-type subunits of the plant SnRK1 complexes

**Figure 1:**
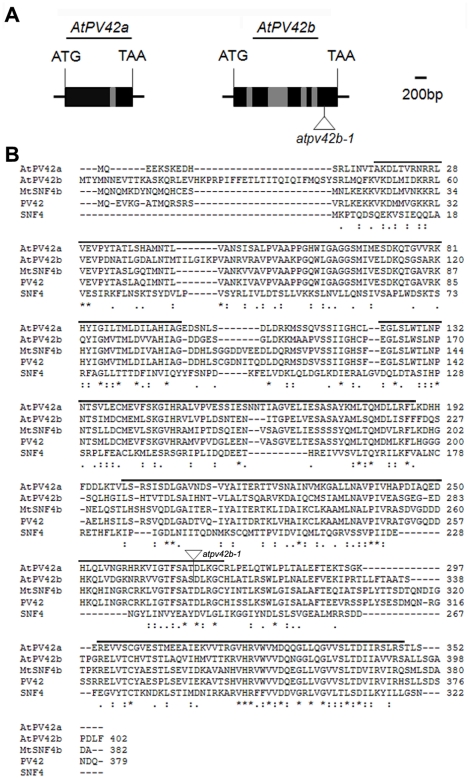
Sequence analysis of *AtPV42a* and *AtPV42b*. (A) Gene structures of *AtPV42a* and *AtPV42b*. A triangle indicates the T-DNA insertion site in *atpv42b-1* (CS823876). Black boxes, grey boxes, and lines represent exons, introns, and untranslated regions, respectively. (B) Alignment of CBS-domain containing proteins from plants including *Arabidopsis* (*At*), *Phaseolus vulgaris* (*Pv*), and *Medicago truncatula* (*Mt*), and yeast (*Saccharomyces cerevisiae*). Identical residues are marked with asterisks. Conserved and semi-conserved substitutions are denoted by ‘:’ and ‘.’, respectively. The overlined CBS domains were predicted using Pfam in the following website (http://www.sanger.ac.uk/Users/agb/CBS/CBS.html). A triangle indicates the position of the T-DNA insertion in *atpv42b-1*.

*AtPV42a* (*At1g15330*) and *AtPV42b* (*At1g80090*) genes are two close homologues in *Arabidopsis*. The *AtPV42a* gene consists of 2 exons and 1 intron, while *AtPV42b* consists of 5 exons and 4 introns (Figure 1A). A BLAST search against the NCBI protein database revealed that AtPV42a and AtPV42b were two *Arabidopsis* proteins homologous to PV42 from *Phaseolus vulgaris*, which is a founding member of the PV42 class of γ-type subunits of the plant SnRK1 complexes [3], [23]. AtPV42a and AtPV42b shared 60% and 54% amino acid identity with PV42, respectively. Multiple sequence alignment and protein domain analysis revealed that like other γ-type subunits of the SNF1/AMPK/SnRK1 complexes, such as SNF4 from yeast, AtPV42a and AtPV42b contained four CBS domains (Figure 1B). These sequence analyses imply that both AtPV42a and AtPV42b are putative members in the PV42 class of γ-type subunits of the *Arabidopsis* SnRK1 complex.

### Expression of *AtPV42a* and *AtPV42b* in *Arabidopsis*

**Figure 2:**
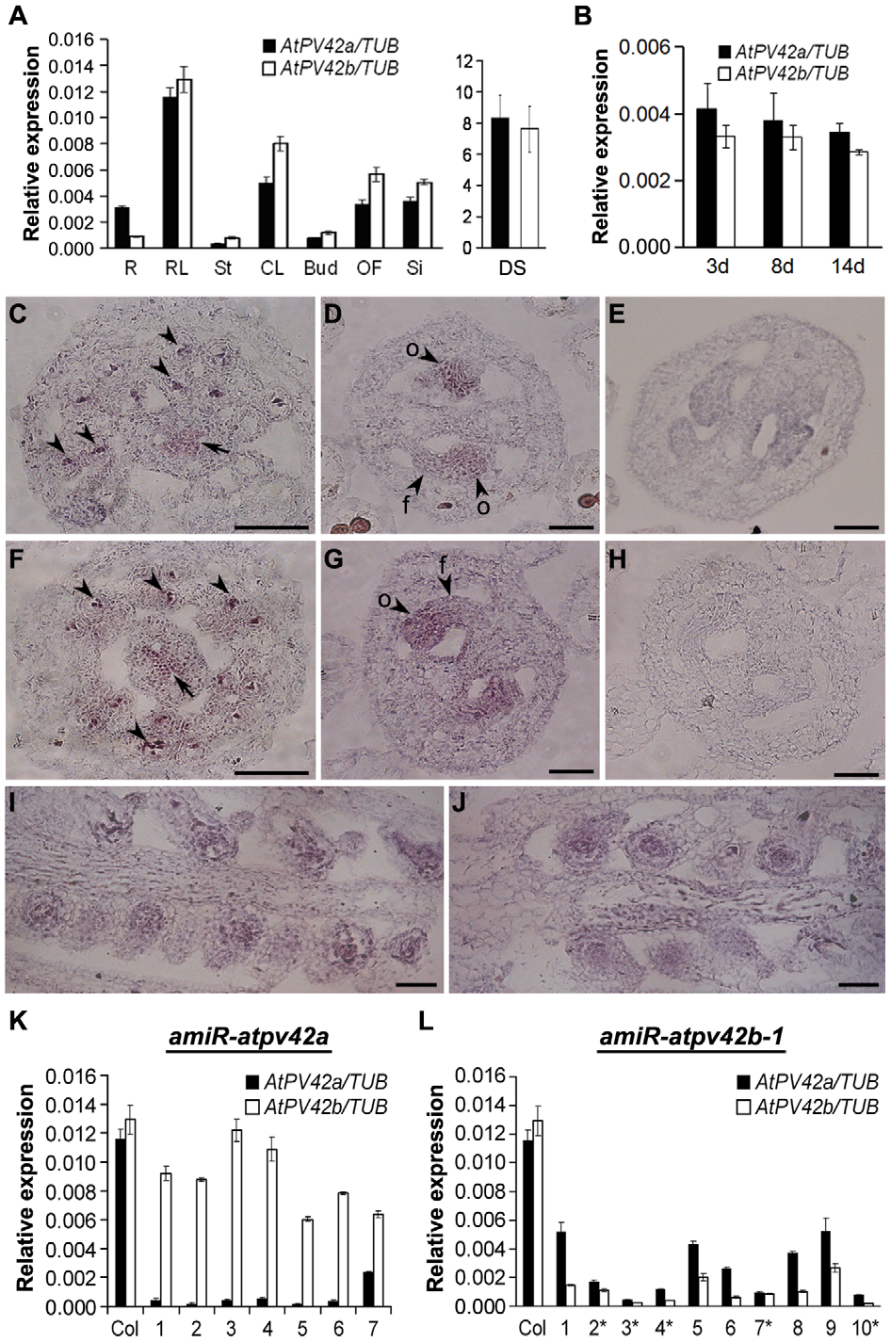
Expression of *AtPV42a* and *AtPV42b* in wild-type and transgenic plants. (A) Transcript levels of *AtPV42a* and *AtPV42b* in various tissues from 28-day-old adult plants and dry seeds of Col wild-type. R, root; RL, rosette leaf; St, stem; CL, cauline leaf; Bud, unopen floral bud; OF, open flower; Si, silique; DS, dry seed. (B) Transcript levels of *AtPV42a* and *AtPV42b* in the seedlings 3, 8, and 14 days after germination. (C-J) In situ hybridization of *AtPV42a* and *AtPV42b* in wild-type developing flowers. (C, F) Transverse section of a stage 9 flower hybridized with the antisense *AtPV42a* (C) or *AtPV42b* (F) probe. Arrows indicate the labelled septum inside the gynoecia, while arrowheads indicate some labelled microspore mother cells in the locules. Bars, 100 µm. (D, E, G, H) Transverse section of a gynoecium from a stage 11 flower hybridized with the antisense (D) or sense probe (E) of *AtPV42a* or the antisense (G) or sense probe (H) of *AtPV42b*. f, funiculus; o, ovule. Bars, 50 µm. (I, J) Longitudinal section of a gynoecium from a stage 13 flower hybridized with the antisense *AtPV42a* (I) or *AtPV42b* (J) probe. Bars, 70 µm. (K) Transcript levels of *AtPV42a* and *AtPV42b* in rosette leaves of 7 selected *amiR-atpv42a* independent transgenic lines at the T1 generation. (L) Transcript levels of *AtPV42a* and *AtPV42b* in rosette leaves of 10 selected *amiR-atpv42b-1* independent transgenic lines at the T1 generation. Asterisks indicate the transgenic lines showing the low fertility phenotype as shown in Figure 3A, C. Transcript levels in (A, B, K, L) were determined by real-time PCR and are shown relative to *TUB2* expression. Values are the mean ± standard deviation from three replicates.

To examine the spatial and temporal expression patterns of *AtPV42a* and *AtPV42b* in *Arabidopsis*, real-time PCR analyses were performed with gene-specific primers using total RNA extracted from various tissues in 28-day-old adult plants and from the whole seedlings at different developmental stages (3-, 8-, 14-day-old). Overall, *AtPV42a* and *AtPV42b* exhibited a similar spatial expression pattern in most of tissues examined in adult plants (Figure 2A). Their expression was relatively high in rosette leaves, cauline leaves, open flowers, and developing silique, but low in stems and floral buds. The only discrepancy was that the relative expression of *AtPV42b* in roots as compared with other tissues was lower than that of *AtPV42a*. It is noteworthy that both *AtPV42a* and *AtPV42b* were expressed at the highest levels in dry seeds (Figure 2A). In the seedlings 3, 8, and 14 days after germination, the expression of both genes remained at stable levels, with a slight decrease in transcripts levels concomitant to an increase in seedling age (Figure 2B).

We further performed in situ hybridization to study the expression of these two genes in developing flowers. Both genes were detectable in the developing septum inside the gynoecia and microspore mother cells in the locules of stage 9 flowers (Figure 2C, F). Their expression levels were either low or absent in anther cells in the flowers at late stages (Figure S1), but persistent in developing ovules in stages 11 and 13 flowers. In stage 11 flowers in which integuments were just initiated on the ovules, the expression of both genes was detected in funiculi and ovules (Figure 2D, E, G, H). In stage 13 flowers in which integuments completely enveloped the nucellus, both genes were also expressed in whole ovules (Figure 2I, J).

High sequence similarity and comparable gene expression patterns between *AtPV42a* and *AtPV42b* indicate that they may play similar roles in *Arabidopsis* growth and development.

### Artificial microRNA-mediated silencing of *AtPV42a* and *AtPV42b*

To investigate the biological roles of *AtPV42a* and *AtPV42b* in *Arabidopsis*, we first attempted to identity insertion mutants from public resources. While insertion mutants of *AtPV42a* were not available in all public resources searched, a SAIL line (CS823876) containing a T-DNA insertion at the last exon of the *AtPV42b* gene was obtained from Arabidopsis Biological Resource Center and named as *atpv42b-1* (Figure 1A). The T-DNA insertion and the resulting disrupted transcription of *AtPV42b* in *atpv42b-1* were confirmed by genotyping PCR and RT-PCR using the primers flanking the insertion site, respectively (Figure S2, Figure S3). *atpv42b-1* did not exhibit visible phenotypes under normal growth conditions. This could be due to two reasons: (1) the incomplete *AtPV42b* transcript produced from the coding region preceding the T-DNA insertion site may still function; (2) functional redundancy between *AtPV42a* and *AtPV42b* may exist.

**Figure 3:**
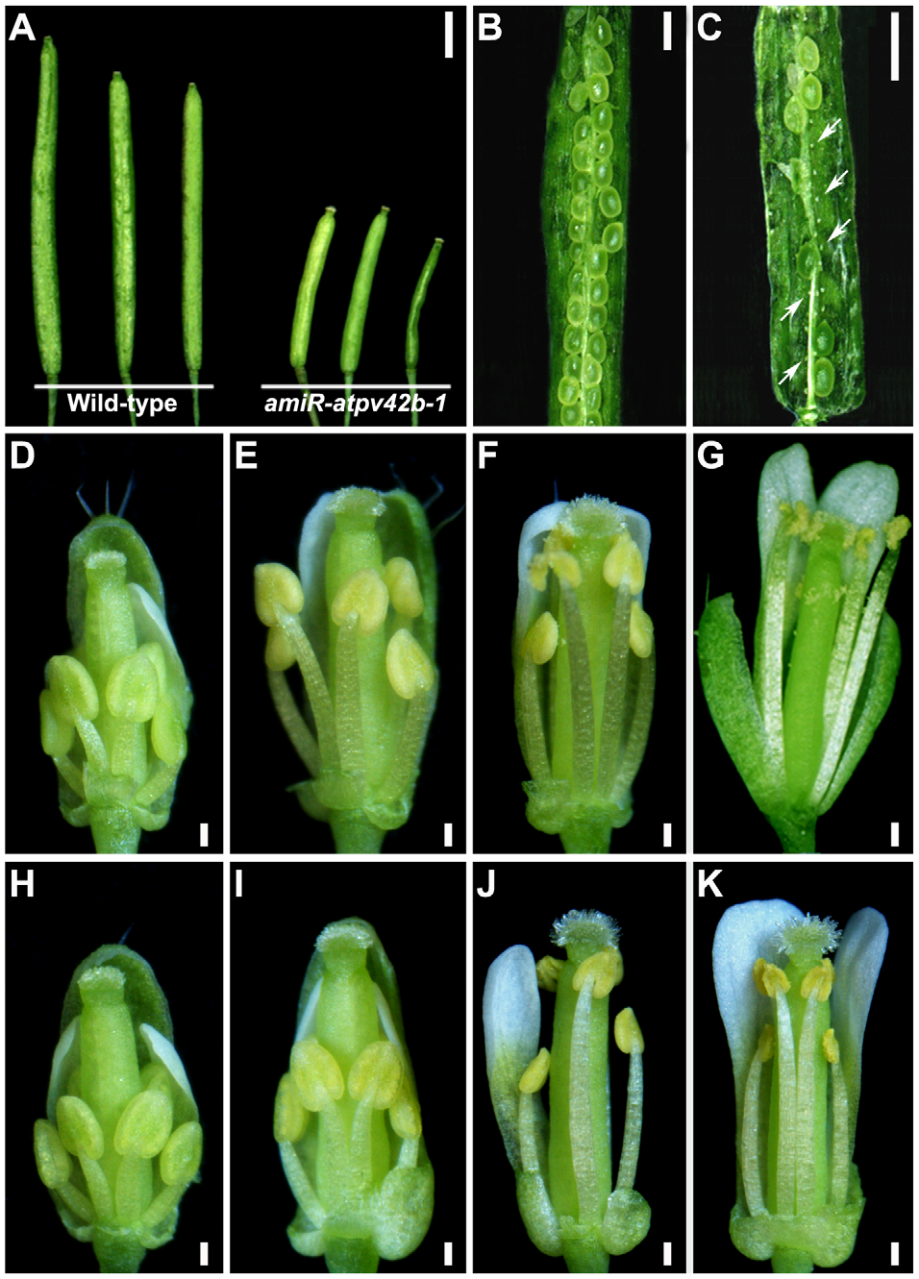
Phenotypes of siliques and developing flowers in wild-type and *amiR-atpv42b-1*(line 10). (A) Comparison of fully grown siliques from wild-type and *amiR-atpv42b-1* plants. (B) A wild-type silique with full seed set. (C) An *amiR-atpv42b-1* silique with reduced seed set and undeveloped ovules, some of which are indicated by arrows. (D-G) Wild-type flowers at early stage 12 (D), late stage 12 (E), stage 13 (F) and stage 14 (G). (H-K) *amiR-atpv42b-1* flowers at early stage 12 (H), late stage 12 (I), stage 13 (J) and stage 14 (K). Flowers at stage 14 show delayed filament elongation and significantly reduced production of pollen grains. Bars in (A–C), 1 mm; Bars in (D, E, H, and I), 150 µm; Bars in (F, G, J, and K), 200 µm.

To create knockdown lines for *AtPV42a* and *AtPV42b*, we generated *amiR-atpv42a* and *amiR-atpv42b-1* transgenic plants that expressed artificial microRNAs specifically targeting these two respective genes. We obtained 17 and 20 independent transformants for *amiR-atpv42a* and *amiR-atpv42b-1* at the T1 generation, respectively. None of the 17 *amiR-atpv42a* transgenic lines showed visible phenotype, whereas 9 *amiR-atpv42b-1* lines developed shorter siliques and reduced seed sets (Figure 3A–C). Since *AtPV42a* and *AtPV42b* shared high sequence similarity, microRNAs designed for either of them could simultaneously affect the expression of the other in transgenic plants. Thus, the expression of both *AtPV42a* and *AtPV42b* was examined in rosette leaves of 7 and 10 selected *amiR-atpv42a* and *amiR-atpv42b-1* T1 transformants, respectively. As expected, the expression levels of *AtPV42a* were dramatically decreased, whereas the levels of *AtPV42b* were not significantly changed in the 7 *amiR-atpv42a* transformants (Figure 2K). However, in most of the 10 selected *amiR-atpv42b-1* lines, the transcript levels of both *AtPV42a* and *AtPV42b* were significantly downregulated, and such downregulation was relevant to the low fertility phenotype exhibited by these transgenic lines (Figure 2L, Table 1). These observations demonstrate that plants are defective in reproductive development only when the expression of both *AtPV42a* and *AtPV42b* is significantly compromised, suggesting that *AtPV42a* and *AtPV42b* function redundantly in controlling *Arabidopsis* reproductive development. Therefore, we chose the transgenic line 10 of *amiR-atpv42b-1* (hereafter referred to as *amiR-atpv42b-1*) (Figure 2L, Table 1), which displayed the strongest phenotypes, for further morphological and molecular characterization.

**Table 1:** Phenotypic analysis of wild-type, *amiR-atpv42b-1*, and *amiR-atpv42b-2* seeds.

	Normal	Unfertilized	Aborted
Col	467 (92.8%)	29 (5.8%)	7 (1.4%)
*amiR-atpv42b-1* (line 2)	203 (40.0%)	297 (58.6%)	7 (1.4%)
*amiR-atpv42b-1* (line 3)	191 (35.1%)	331 (60.8%)	22 (4.0%)
*amiR-atpv42b-1* (line 10)	74 (14.3%)	443 (85.4%)	2 (0.4%)
*amiR-atpv42a atpv42b-1*	259(57.3%)	187(41.4%)	6(1.3%)
*amiR-atpv42b-2* (line 4)	483 (91.7%)	31 (5.9%)	13 (2.5%)

For each genotype, the phenotype was scored using fully grown siliques from at least 10 plants.

To confirm the functional redundancy of *AtPV42a* and *AtPV42b*, the transgenic line 3 of *amiR-atpv42a* (hereafter referred to as *amiR-atpv42a*) (Figure 2K) was crossed with the T-DNA insertion mutant *atpv42b-1* (Figure 1A). The resulting homozygous progenies, where the expression of both *AtPV42a* and *AtPV42b* is disrupted (Figure S4), exhibited a similar low fertility phenotype to *amiR-atpv42b-1* (Table 1). This is in agreement with our suggestion that *AtPV42a* and *AtPV42b* function redundantly in reproductive development.

To further test whether a single knockdown of *AtPV42b* impairs the reproductive process, we generated *amiR-atpv42b-2* transgenic plants overexpressing another artificial microRNA targeting *AtPV42b*. Real-time PCR assay showed that in 6 selected *amiR-atpv42b-2* independent lines, *AtPV42b* was significantly downregulated, whereas the expression of *AtPV42a* was almost unaffected (Figure S5). Unlike *amiR-atpv42b-1*, *amiR-atpv42b-2* transgenic plants were morphologically indistinguishable from wild-type plants (Table 1). These results, together with the phenotype of *atpv42b-1*, further corroborate that *AtPV42b* functions redundantly with other factors, such as *AtPV42a*, to regulate reproductive development.

We also created 18 and 15 independent transgenic lines overexpressing *AtPV42a* and *AtPV42b*, respectively. While *AtPV42a* and *AtPV42b* were overexpressed in most of these lines, none of them exhibited abnormal phenotypes in reproductive development (data not shown).

### *amiR-atpv42b-1* is defective in late stamen development

To uncover the developmental events responsible for the reduced fertility in *amiR-atpv42b-1,* we compared the morphology of *amiR-atpv42b-1* and wild-type plants at different developmental stages. *amiR-atpv42b-1* appeared normal during the vegetative phase, floral transition, and early stages of flower development (data not shown). In *amiR-atpv42b-1* floral buds at stage 12, floral organs were still morphologically normal (Figure 3D, E, H, I), whereas at the anthesis stage (flower stages 13 and 14), fewer pollen grains were produced (Figure 3F, G, J, K). Moreover, the filaments in *amiR-atpv42b-1* did not elongate enough to position the locules above the stigma at the flower stage 14 (Figure 3G, K).

**Figure 4:**
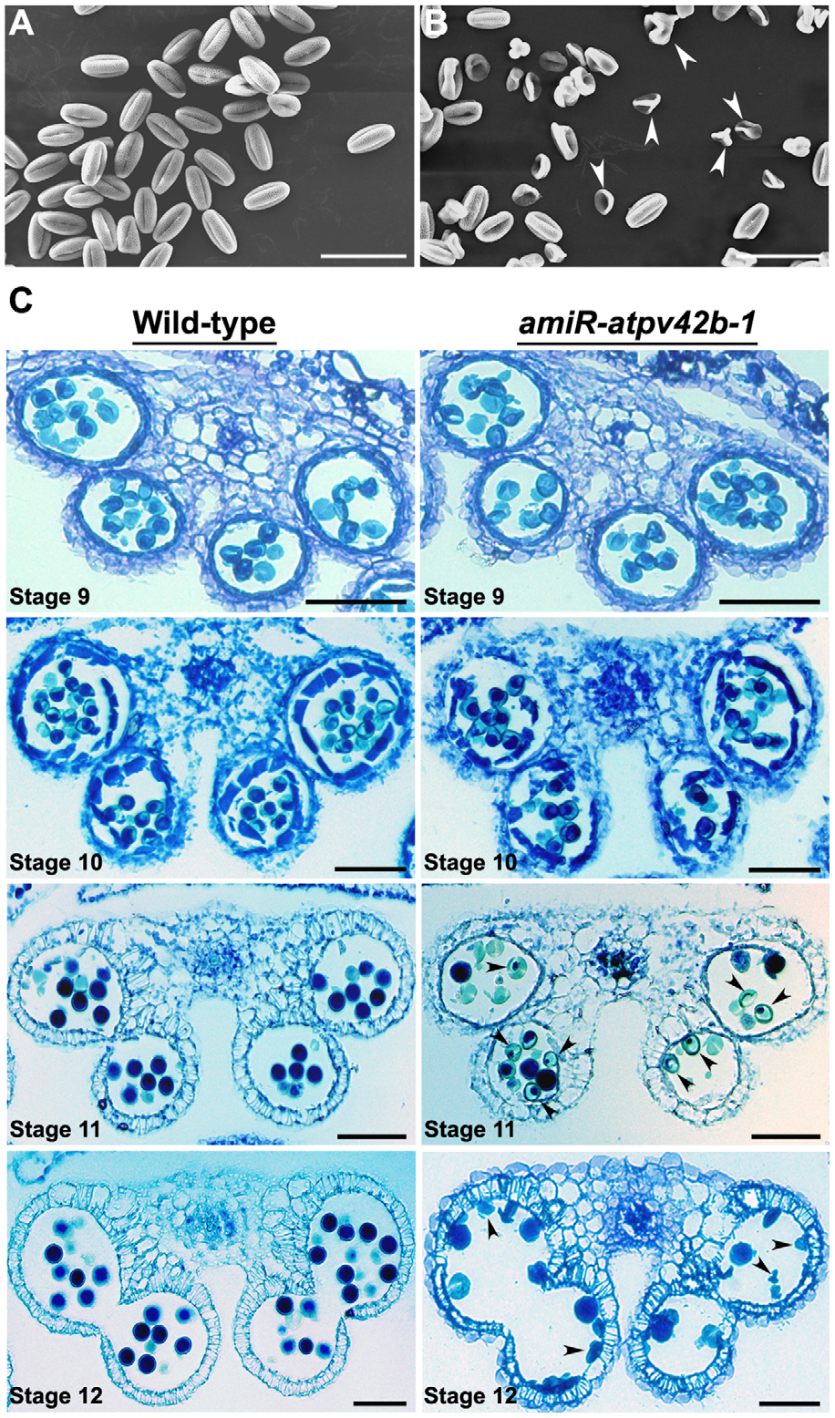
Pollen grain development in wild-type and *amiR-atpv42b-1.* (A) Scanning electron micrograph (SEM) of mature pollen grains collected from wild-type flowers at stages 13–14. (B) SEM of pollen grains collected from *amiR-atpv42b-1* flowers at stages 13–14. The majority of the pollen grains are shrunken and exhibit a collapsed morphology (arrowheads). (C) Transverse sections of wild-type and *amiR-atpv42b-1* anthers at anther stages 9, 10, 11, and 12. Arrowheads indicate defective pollen grains in *amiR-atpv42b-1* anthers. Bars, 50 µm.

Scanning electron micrograph (SEM) revealed that as compared with those in wild-type plants, more than 50% of the pollen grains from *amiR-atpv42b-1* were severely shrunken and of irregular shapes at the anthesis stage (Figure 4A, B, Table S1). Thus, we examined the transverse sections of wild-type and *amiR-atpv42b-1* anthers under the light microscope to investigate the changes at the cellular level. We found that even at anther stage 10 (approximately flower stages 11–12) when tapetum degeneration initiated, there was no visible difference in microspores and other anther tissues between wild-type and *amiR-atpv42b-1* (Figure 4C), indicating that microsporogenesis in *amiR-atpv42b-1* may be normal [30]. However, at anther stage 11 when pollen mitotic divisions occur in wild-type plants, most of the microspores in *amiR-atpv42b-1* seemed to lack dense cell contents (Figure 4C). At anther stage 12, when microgametogenesis was completed and microspores developed into tricellular haploid pollen grains, many pollen grains in *amiR-atpv42b-1* anthers were shrunken with irregular shapes (Figure 4C), which is consistent with the SEM results (Figure 4A, B).

**Figure 5:**
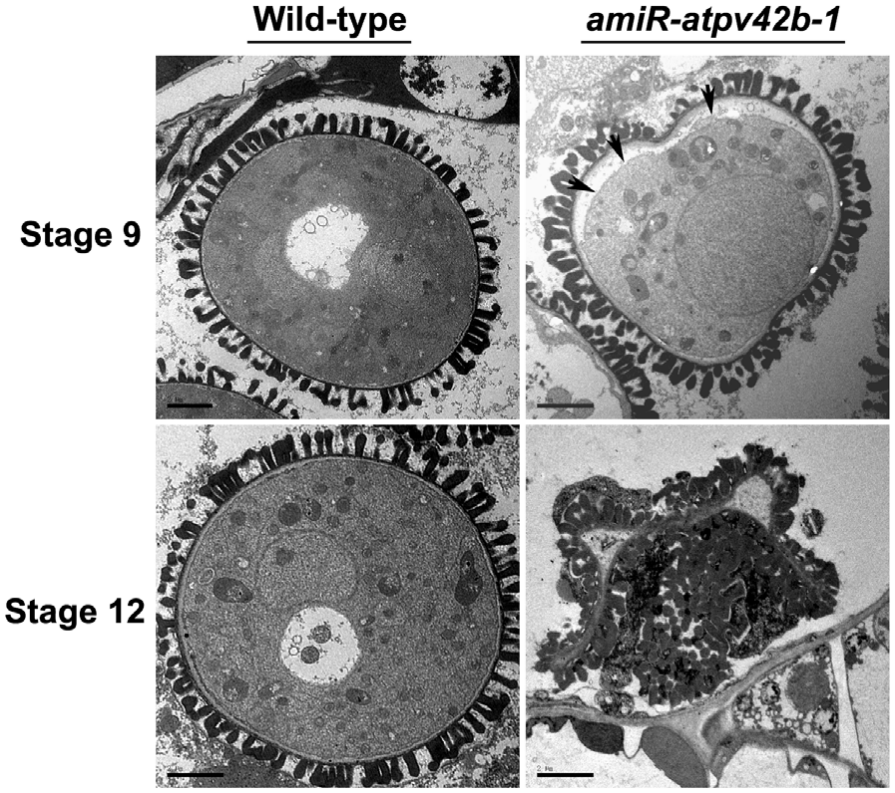
Transmission electron microscopy of wild-type and *amiR-atpv42b-1* pollen grains. Wild-type and *amiR-atpv42b-1* pollen grains at anther stages 9 and 12 are shown. In *amiR-atpv42b-1,* during plasmolysis occurring at anther stage 9, the plasma membrane (arrows) of the pollen grain is withdrawn from the cell wall, while at anther stage 12, the cytoplasm is almost invisible in the severely shrunken pollen grain. Bars, 2 µm.

We further performed transmission electron microscopy (TEM) to compare the finer cellular structures of the developing pollen cells in wild-type and *amiR-atpv42b-1* anthers. Although the transverse sections of *amiR-atpv42b-1* anthers at anther stage 9 showed normal-looking microspores under the light microscope (Figure 4C), TEM revealed that the plasma membrane was often withdrawn from the cell wall in *amiR-atpv42b-1* pollen grains (Figure 5). This is an indication of plasmolysis, implying the abnormal cellular osmotic homeostasis inside the *amiR-atpv42b-1* pollen grains. At anther stage 12, shrunken pollen grains in *amiR-atpv42b-1* lost most of the cell contents (Figure 5), indicating that there is a continuous improper cellular osmotic homeostasis from anther stage 9 to 12. These TEM results explain why the microspores at anther stage 11 in *amiR-atpv42b-1* demonstrate reduced amount of cell contents (Figure 4C).

Taken together, these observations suggest that downregulation of *AtPV42a* and *AtPV42b* in *amiR-atpv42b-1* results in the production of some collapsed pollen grains particularly at late stages of anther development. This might partly contribute to the reduced fertility observed in *amiR-atpv42b-1.*

### Female gametophytes of *amiR-atpv42b-1* are defective in pollen tube reception

**Figure 6:**
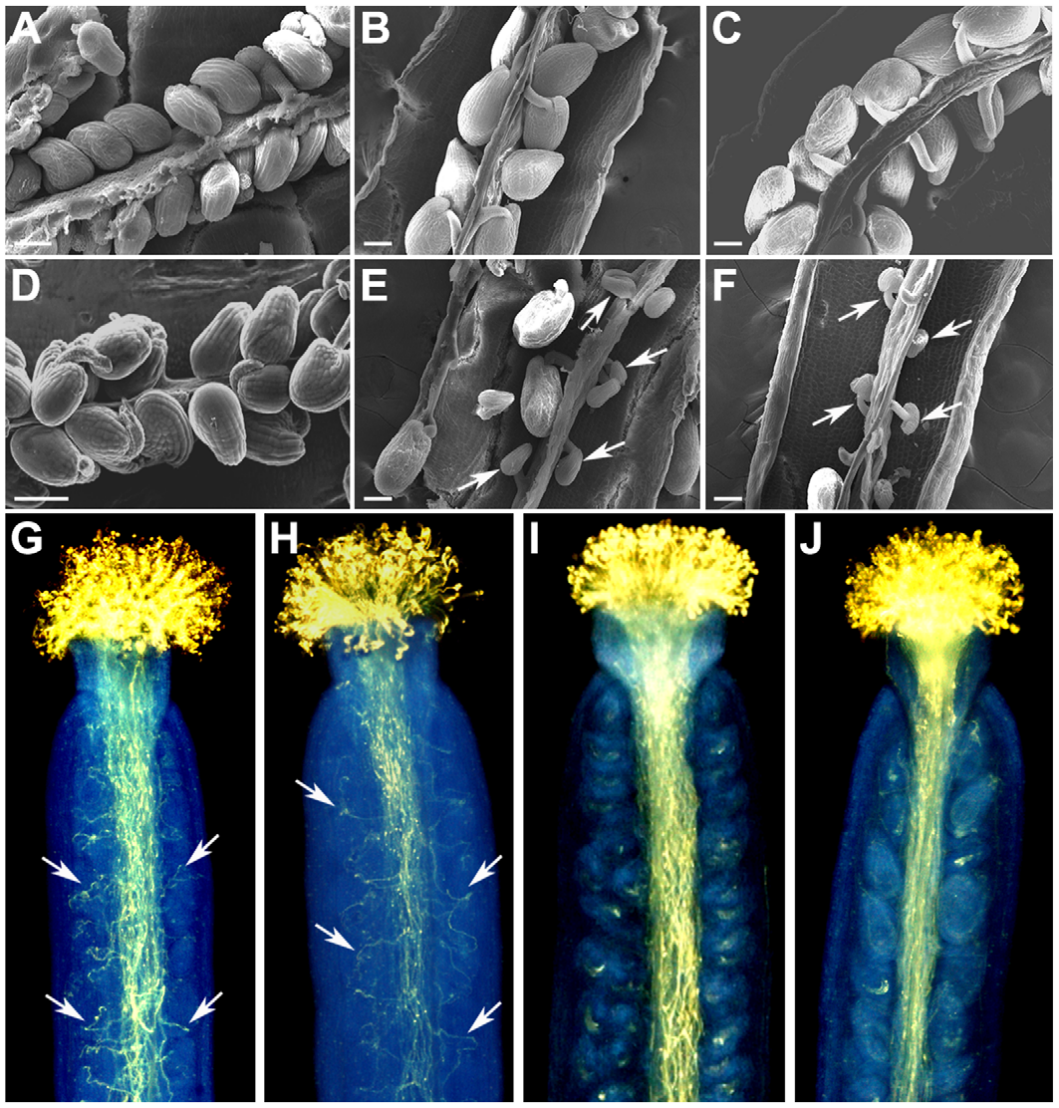
Phenotypic analyses of reciprocal crosses between wild-type and *amiR-atpv42b-1* plants. (A) SEM of wild-type ovules at flower stage 12 prior to fertilization. (B, C) SEMs of developing seeds 2 days after pollination from the following reciprocal crosses: Col ♀×Col ♂ (B) and Col ♀× *amiR-atpv42b-1* ♂ (C). (D) SEM of *amiR-atpv42b-1* ovules at flower stage 12 prior to fertilization. (E, F) SEMs of developing seeds 2 days after pollination from the following reciprocal crosses: *amiR-atpv42b-1* ♀×Col ♂ (E) and *amiR-atpv42b-1* ♀× *amiR-atpv42b-1* ♂ (F). Arrows indicate the unfertilized ovules. (G–J) Aniline blue staining of pollen tube growth inside pistils collected 20 hours after pollination from the following reciprocal crosses: Col ♀×Col ♂ (G), Col ♀×*amiR-atpv42b-1* ♂ (H), *amiR-atpv42b-1* ♀×Col ♂ (I), and *amiR-atpv42b-1* ♀× *amiR-atpv42b-1* ♂ (J). Arrows indicate the lateral growth of pollen tubes. Bars in (A-F), 100 µm.

To determine whether the defective stamen development is the only cause of the low fertility found in *amiR-atpv42b-1,* reciprocal crosses between *amiR-atpv42b-1* and wild-type plants were performed. After saturated pollination with either wild-type or *amiR-atpv42b-1* pollen grains, the majority of wild-type ovules were normally fertilized (Table 2). However, more than 50% of *amiR-atpv42b-1* ovules were unfertilized regardless of the father plants (Table 2). These results indicate that female tissues in *amiR-atpv42b-1* are partly defective. As the morphology of *amiR-atpv42b-1* carpels during flower development was almost normal (Figure 3H-K), we then examined the ovules inside the carpels before and after fertilization using SEM. *amiR-atpv42b-1* ovules were morphologically comparable to wild-type ones prior to fertilization (Figure 6A, D). SEM of wild-type pistils two days after saturated pollination with either wild-type or *amiR-atpv42b-1* pollen grains revealed that most of the ovules have been fertilized (Figure 6B, C), which is in agreement with the observation on seed sets in wild-type plants (Table 2). This implies that some healthy pollen grains produced in *amiR-atpv42b-1* could function normally during fertilization. On the contrary, many *amiR-atpv42b-1* ovules were not fertilized after saturated pollination regardless of the source of pollen grains (Figure 6E, F), demonstrating the fertilization defect in *amiR-atpv42b-1* female gametophytes.

**Table 2:** Phenotypic analysis of reciprocal crosses between wild-type and *amiR-atpv42b-1* plants.

Ovule × Pollen	Normal	Unfertilized	Aborted
Col × Col	254 (92.4%)	7 (2.5%)	14 (5.1%)
Col × *amiR-atpv42b-1*	245 (85.4%)	37 (12.9%)	5 (1.7%)
*amiR-atpv42b-1* × Col	146 (46.8%)	160 (51.3%)	6 (1.9%)
*amiR-atpv42b-1* × *amiR-atpv42b-1*	133 (43.8%)	158 (52.0%)	13 (4.3%)

Flowers were emasculated and fertilized manually with pollen. The phenotype was examined one week after fertilization.

We further investigated the growth of pollen tubes inside the pistils after reciprocal crosses to explore the underlying mechanism of the fertilization defect in *amiR-atpv42b-1.* At 20 hours after saturated pollination, pistils were collected, fixed, cleared, stained with aniline blue, and observed under UV microscope (Figure 6G–J). Likewise, the pollen tube growth was independent of the source of pollen grains, but mainly relied on the maternal plants used. In wild-type pistils pollinated with either wild-type or *amiR-atpv42b-1* pollen grains, pollen tubes elongated longitudinally through the stylar transmitting tract from the stigma, and then elongated laterally to the ovules for fertilization (Figure 6G, H). However, in *amiR-atpv42b-1* pistils, although the longitudinal pollen tube growth through the transmitting tract was similar to that in wild-type pistils, the lateral pollen tube growth towards the ovules was largely abolished (Figure 6I, J). These results demonstrate that the fertilization defect in *amiR-atpv42b-1* pistils is mainly due to abnormal pollen tube attraction by ovules.

As synergid cells are responsible for guiding pollen tubes to the embryo sac by secreting chemical attractants [31], [32], we tested the expression of several synergid cell-specific genes in *amiR-atpv42b-1* and found that the expression of *LORELEI* (*LRE*) was altered (Figure S6). It has been reported that *LRE* is involved in proper pollen tube reception [33]. In *lre* mutants, pollen tubes reaching the embryo sac frequently continue to grow inside the embryo sac, resulting in the failure of fertilization. In *amiR-atpv42b-1,* where pollen tube attraction was blocked, the expression level of *LRE* was significantly increased in open flowers (Figure S6), indicating that disruption of pollen tube guidance to the embryo sac in *amiR-atpv42b-1* might be relevant to the upregulation of *LRE*.

## Discussion

Plant SnRK1-type kinases, which serve as the catalytic α-subunits and are associated with non-catalytic β-type and γ-type subunits in the SnRK1 complexes, play important roles in the global regulation of metabolism in response to cellular and environmental signals [1]. Studies in a wide range of plant species have shown that SnRK1s are involved in the regulation of various developmental processes. For example, disruption of the SnRK1 kinase in transgenic barley plants results in abnormal pollen development and male sterility [34]. The rice SnRK1A protein kinase acts as an important intermediate in the sugar signaling cascade and plays a key role in regulating seed germination and seedling growth [35]. In the moss *Physcomitrella patens*, downregulation of SnRK1 kinases shows pleiotropic phenotypes including developmental abnormalities [36]. Although these studies have shed light on the function of the catalytic α-subunits in the SnRK1 complexes, the biological function of γ-type subunits and their effects on plant development are so far unknown.

In this study, we have characterized *AtPV42a* and *AtPV42b*, two close homologues encoding CBS domain-containing proteins in the PV42 class of γ-type subunits of the SnRK1 complexes in *Arabidopsis*. Downregulation of *AtPV42a* and *AtPV42b* not only disturbs late stamen development, but also impairs pollen tube reception conferred by the female gametophyte, which eventually results in the low fertility. The function of *AtPV42a* and *AtPV42b* in the reproductive development is consistent with their expression in floral organs. Both genes are expressed in the developing septum inside the gynoecia and microspore mother cells in stage 9 flowers, and later in developing ovules. These results suggest that the non-catalytic γ-type subunits in the SnRK1 complexes play an important role in mediating plant reproductive growth. This is in accordance with a previous finding that shows the involvement of an α-type subunit of the SnRK1 complexes in pollen development in barley [34].

In *amiR-atpv42b-1* where *AtPV42a* and *AtPV42b* are downregulated, defective stamen development is mainly attributed to abnormal microgametogenesis. Histological analysis of anther transverse sections and SEM and TEM of pollen cells demonstrate that anther defects in *amiR-atpv42b-1* mainly occur in microspores at the mitotic phase. Pollen development is divided into two phases, microsporogenesis and microgametogenesis [37]–[39]. During microsporogenesis, pollen mother cells undergo meiosis and form tetrads of microspores within the pollen sacs. During microgametogenesis, separate microspores undergo mitosis and differentiate into mature pollen grains. In this process, enzymes are abundant and metabolism is highly active in microspores, which obtain nutrients and water from degenerated tapetum and undergo an asymmetric mitotic division to generate a large vegetative cell and a small generative cell. The generative cell further undergoes a second mitosis to form two generative cells. It is noteworthy that active carbohydrate metabolism not only provides energy for cell division and differentiation during microgametogenesis, but also stores essential substances required for subsequent pollen germination and pollen tube growth. As the SnRK1 complex is the global regulator of carbohydrate metabolism [40], it may also be involved in microgametogenesis. This hypothesis is partly supported by our observation that disruption of *AtPV42a* and *AtPV42b*, which encode the γ-type subunits of the SnRK1 complexes, compromises microgametogenesis, thus resulting in abnormal pollen grains.

*amiR-atpv42b-1* also exhibits impaired pollen tube attraction by the female gametophyte. In *Arabidopsis*, synergid cells accompanying the egg cell are primarily responsible for the pollen tube guidance to the female gametophyte and the release of sperm cells [31], [32], [41]. Intracellular metabolism is highly active in synergid cells, which uptake and transport metabolites into the embryo sac, and secrete some chemical substances into the filiform apparatus that is involved in pollen tube guidance and reception [42]–[45]. So far the molecular mechanisms underlying the function of synergid cells still remain largely unknown. Several genes specifically expressed in synergid cells, including *MYB98*, *ZmEA1*, and *LRE*, have been suggested as important regulators for pollen tube guidance [32], [33], [43]. Our results have shown that *LRE* is upregulated in *amiR-atpv42b-1,* which might be partially responsible for the defect in pollen tube attraction. *LRE* encodes a putative plant-specific GPI-anchor protein (GAP), a eukaryotic protein that functions as a lipid raft in cell-cell signalling. *LRE* is required for proper pollen tube guidance to the embryo sac in *Arabidopsis* and silencing of *LRE* results in continuous pollen tube growth inside the embryo sac [33]. It is possible that the SnRK1 complex plays a role in the intracellular metabolism of synergid cells. Thus, downregulation of *AtPV42a* and *AtPV42b* may affect normal metabolism of synergid cells, which is relevant to the altered expression of key regulators, such as *LRE*. This eventually results in the impaired pollen tube attraction by the female gametophyte.

Taken together, our results suggest that *AtPV42a* and *AtPV42b* play redundant roles in regulating male gametogenesis and pollen tube guidance. It will be interesting to further investigate how they interact with other subunits of the SnRK1 complex to regulate metabolic responses and contribute to the regulation of *Arabidopsis* development. In addition, the highest expression levels of *AtPV42a* and *AtPV42b* are detected in dry seeds (Figure 2A). It has been reported that disruption of the α-type subunit of the SnRK1 complex retards seed germination in rice [35]. Thus, besides the roles in regulating microgametogenesis and pollen tube guidance, *AtPV42a* and *AtPV42b* might also play a role in mediating the function of SnRK1 complexes in post-fertilization processes, such as seed development. This will be another intriguing topic to be further studied.

## Materials and Methods

### Plant materials and growth conditions

All *Arabidopsis* plants used in this study were in the Columbia (Col-0) background. For plants grown on soil, seeds were stratified at 4°C for 2 to 3 days and then transferred onto compost soil in shallow trays. The trays were placed in an environmentally controlled growth room at 22°C with a light cycle of 16 h light/8 h dark.

### Plasmid construction

The artificial microRNAs (amiRNAs) and the primers for subsequent cloning were designed according to the procedures and criteria on the website (http://wmd3.weigelworld.org/cgi-bin/webapp.cgi) [46] using the miR319a precursor-containing plasmid pRS300 as a template. Predicated mature miRNA sequences were 5′-UGAAAACGUACCUAUCACUUC-3′ for *amiR-atpv42a*, 5′-UGAAUAGUCAUAGUGUUCAGG-3′ for *amiR-atpv42b-1*, and 5′-UUACUGUCCAAUGGGACCGAU -3′for *amiR-atpv42b-2*. Primers used in the construction of *amiR-atpv42a* and *amiR-atpv42b-1* are listed in Table S2. The final PCR products were digested with *Eco*RI and *Bam*HI, and then cloned into the corresponding sites of the pGreen0229-35S binary vector [47].

To construct *35S:AtPV42a*, the coding region of *AtPV42a* was amplified using the primers 5′-ATAGAATTCAACAGTAGTAGAACACTATGCAAG-3′ and 5′- GAAACTAGTGATTACGAAAGAGTAGATCTTAGG-3′. Similarly, to construct *35S:AtPV42b*, the coding region of *AtPV42b* was amplified using the primers 5′-GAACTGCAG ATGACATATATGAATAATGAAG-3′ and 5′-AATACTAGTTTGCGAGTTAAAACAGATCC-3′. The PCR products were digested and cloned into the pGreen0229-35S binary vector [47].

### Arabidopsis transformation

*Agrobacterium tumeficians* GV3101 was used for floral dipping according to the published method with minor modifications [48]. Transgenic plants were screened for herbicide resistance against Basta (300 mg/l).

### Expression analysis

Total RNA from different organs was isolated using the FavorPrep^TM^ Plant Total RNA Mini Kit (Favorgen) and reverse transcribed using the SuperScript™ RT-PCR System (Invitrogen). Real-time PCR was carried out using the Power SYBR® Green PCR Master mix (Applied Biosystems) as previously reported [49]. Primers used in real-time PCR amplifications are listed in Table S2. Non-radioactive in situ hybridization and synthesis of RNA probes were carried out as previously published [49].

### Aniline blue staining of pollen tube growth

Aniline blue staining of pollen tube growth in pistils was carried out as previously described [50].

### Histological analysis

Inflorescence apices of wild-type and *amiR-atpv42b-1* plants were collected, fixed overnight in the fixative solution (2.5% (v/v) glutaraldehyde in 1×PBS), dehydrated using increasing concentrations of ethanol (30%, 40%, 50%, 60%, 70%, 85%, 95%, 4×100%), embedded in histowax, and transverse-sectioned (8 µm) using a microtome. The transverse sections were deparaffinised by histoclear and rehydrated with a graded ethanol series (95%, 90%, 80%, 60%, and 30%). After rinsing with water, the sections were stained in 1% toluidine blue for 1 min and observed under a compound microscope.

### Scanning electron microscopy (SEM)

Mature pollen grains collected from wild-type and *amiR-atpv42b-1* flowers after anther dehiscence were spread onto the surface of adhesive tapes, and directly observed under a JSM-6360LV scanning electron microscope (JEOL) at an accelerating voltage of 20 kV. Pistils were collected 1 to 2 days after pollination, and carefully opened with a sharp needle. SEM of pistils was performed as previously reported with some modifications [51]. Pistils were fixed with FAA (50% ethanol, 3.7% formaldehyde, and 5% acetic acid) for 2 h, and dehydrated through increasing concentrations of ethanol (30%, 50%, 70%, 80%, 90%, and 100%). After replacing absolute ethanol with the transfer liquid (amyl acetate), pistils were critical-point dried using CO_2_, mounted for sputter coating with gold palladium for 25 s, and observed under JSM-6360LV.

### Transmission electron microscopy (TEM)

TEM was performed as previously reported [52]. Wild-type and *amiR-atpv42b-1* inflorescences were collected, fixed overnight with 2.5% (v/v) glutaraldehyde in 1×PBS (0.1 M, pH 7.4), washed with 1×PBS for five times, and post-fixed in 1% OsO4 for 16 h at 4°C. The specimens were subsequently washed with 1×PBS for five times, dehydrated in a graded ethanol series, replaced by epoxy dimethylmethane, and embedded in Epon812 resin. Semi-thin sections 2–4 µm in thickness were obtained using glass knives, stained with 0.5% toluidine blue in 1×PBS, and then examined under a light microscope to confirm the developmental stages of microspores. Selected sections were further cut into sections 80–90 nm in thickness using a diamond knife with Leica Ultra-S microtome, stained with 2% uranyl acetate for 90 min and 6% lead citrate for 15 min, and then observed under a JEM-1230 transmission electron microscope (JEOL) at 90 kV.

## Supporting Information

Figure S1 In situ hybridization of *AtPV42a* and *AtPV42b* in a stage 11 wild-type flower. A transverse section was hybridized with the antisense *AtPV42a* (A) or *AtPV42b* (B) probe. There are hybridization signals inside the gynoecia (arrows), while no signals are detectable in anther cells (arrowheads). Bars, 50 µm.
(TIF)

Figure S2 Genotyping of *atpv42b-1* mutants using PCR analysis. Lanes 1, 3: PCR products amplified with the left (CS823876_LP) and right (CS823876_RP) primers flanking the *AtPV42b* genomic region. Lanes 2, 4: PCR products amplified with the T-DNA left border primer (LB2_SAIL) and the right primer for *AtPV42b* (CS823876_RP). We detected the amplification of a T-DNA fragment (lane 4), but not the *AtPV42b* genomic region (lane 3) in *atpv42b-1*, indicating that *atpv42b-1* is a homozygous T-DNA insertion mutant.
(TIF)

Figure S3 Transcript levels of *AtPV42b* in rosette leaves (RL) and open flowers (OF) of wild-type and *atpv42b-1* plants. *AtPV42b* expression is undetectable in *atpv42b-1* as compared to that in wild-type plants. Transcript levels were determined by real-time PCR using a pair of primers flanking the T-DNA insertion site and are shown relative to *TUB2* expression. Values are the mean ± standard deviation from three replicates.
(TIF)

Figure S4 Transcript levels of *AtPV42a* and *AtPV42b* in rosette leaves of wild-type and *amiR-atpv42a atpv42b-1* plants. The expression of both *AtPV42a and AtPV42b* is very low in *amiR-atpv42a atpv42b-1* as compared to that in wild-type plants. Transcript levels were determined by real-time PCR and are shown relative to *TUB2* expression. Values are the mean ± standard deviation from three replicates.
(TIF)

Figure S5 Transcript levels of *AtPV42a* and *AtPV42b* in rosette leaves of 6 selected *amiR-atpv42b-2* independent transgenic lines at the T1 generation. *AtPV42a* expression is not significantly affected in these transgenic plants as compared to that in wild-type plants, whereas *AtPV42b* expression is greatly decreased. Transcript levels were determined by real-time PCR and are shown relative to *TUB2* expression. Values are the mean ± standard deviation from three replicates.
(TIF)

Figure S6 Expression of *LRE* in open flowers of wild-type and *amiR-atpv42b-1* plants. *LRE* expression is much upregulated in three independent *amiR-atpv42b-1* lines than in wild-type plants. Transcript levels were determined by real-time PCR and are shown relative to *TUB2* expression. Values are the mean ± standard deviation from three replicates.
(TIF)

Table S1 Phenotypic analysis of *amiR-atpv42b-1* pollen grains.
(DOC)

Table S2 Primers used in this study.
(DOC)

## References

[1] Polge C, Thomas M (2007). SNF1/AMPK/SnRK1 kinases, global regulators at the heart of energy control?. Trends Plant Sci.

[2] Halford NG, Hardie DG (1998). SNF1-related protein kinases: global regulators of carbon metabolism in plants?. Plant Mol Biol.

[3] Slocombe SP, Laurie S, Bertini L, Beaudoin F, Dickinson JR (2002). Identification of SnIP1, a novel protein that interacts with SNF1-related protein kinase (SnRK1).. Plant Mol Biol.

[4] Celenza JL, Carlson M (1984). Structure and expression of the SNF1 gene of saccharomyces-cerevisiae.. Mol Cell Biol.

[5] Celenza JL, Carlson M (1986). A yeast gene that is essential for release from glucose repression encodes a protein-kinase.. Science.

[6] Hardie DG (2004). The AMP-activated protein kinase pathway - new players upstream and downstream.. J Cell Sci.

[7] Carling D (2004). The AMP-activated protein kinase cascade - a unifying system for energy control.. Trends Biochem Sci.

[8] Hardie DG, Sakamoto K (2006). AMPK: A key sensor of fuel and energy status in skeletal muscle.. Physiology.

[9] Rolland F, Baena-Gonzalez E, Sheen J (2006). Sugar sensing and signaling in plants: Conserved and novel mechanisms.. Annu Rev Plant Biol.

[10] Alderson A, Sabelli PA, Dickinson JR, Cole D, Richardson M (1991). Complementation of snf1, a mutation affecting global regulation of carbon metabolism in yeast, by a plant protein-kinase Cdna.. Proc Natl Acad Sci U S A.

[11] Muranaka T, Banno H, Machida Y (1994). Characterization of tobacco protein-kinase NPK5, a homolog of Saccharomyces-cerevisiae SNF1 that constitutively activates expression of the glucose-repressible SUC2 gene for a secreted invertase of S. cerevisiae.. Mol Cell Biol.

[12] Takano M, Kajiya-Kanegae H, Funatsuki H, Kikuchi S (1998). Rice has two distinct classes of protein kinase genes related to SNF1 of Saccharomyces cerevisiae, which are differently regulated in early seed development.. Mol Gen Genet.

[13] Bhalerao RP, Salchert K, Bako L, Okresz L, Szabados L (1999). Regulatory interaction of PRL1 WD protein with Arabidopsis SNF1-like protein kinases.. Proc Natl Acad Sci U S A.

[14] Lovas A, Sos-Hegedus A, Bimbo A, Banfalvi Z (2003). Functional diversity of potato SNF1-related kinases tested in Saccharomyces cerevisiae.. Gene.

[15] Bouly JP, Gissot L, Lessard P, Kreis M, Thomas M (1999). Arabidopsis thaliana proteins related to the yeast SIP and SNF4 interact with AKIN alpha 1, an SNF1-like protein kinase.. Plant J.

[16] Vincent O, Townley R, Kuchin S, Carlson M (2001). Subcellular localization of the Snf1 kinase is regulated by specific beta subunits and a novel glucose signaling mechanism.. Genes Dev.

[17] Hedbacker K, Townley R, Carlson M (2004). Cyclic AMP-dependent protein kinase regulates the subcellular localization of Snf1-Sip1 protein kinase.. Mol Cell Biol.

[18] Jiang R, Carlson M (1997). The Snf1 protein kinase and its activating subunit, Snf4, interact with distinct domains of the Sip1/Sip2/Gal83 component in the kinase complex.. Mol Cell Biol.

[19] Celenza JL, Carlson M (1989). Mutational analysis of the Saccharomyces cerevisiae SNF1 protein kinase and evidence for functional interaction with the SNF4 protein.. Mol Cell Biol.

[20] Rutter GA, Xavier GD, Leclerc I (2003). Roles of 5 ′-AMP-activated protein kinase (AMPK) in mammalian glucose homoeostasis.. Biochem J.

[21] Kleinow T, Bhalerao R, Breuer F, Umeda M, Salchert K (2000). Functional identification of an Arabidopsis Snf4 ortholog by screening for heterologous multicopy suppressors of snf4 deficiency in yeast.. Plant J.

[22] Lumbreras V, Alba MM, Kleinow T, Koncz C, Pages M (2001). Domain fusion between SNF1-related kinase subunits during plant evolution.. EMBO Rep.

[23] Gissot L, Polge C, Jossier M, Girin T, Bouly JP (2006). AKIN beta gamma contributes to SnRK1 heterotrimeric complexes and interacts with two proteins implicated in plant pathogen resistance through its KIS/GBD sequence.. Plant Physiol.

[24] Bateman A (1997). The structure of a domain common to archaebacteria and the homocystinuria disease protein.. Trends Biochem Sci.

[25] Bult CJ, White O, Olsen GJ, Zhou L, Fleischmann RD (1996). Complete Genome Sequence of the Methanogenic Archaeon, Methanococcus jannaschii.. Science.

[26] Ishitani R, Sugita Y, Dohmae N, Furuya N, Hattori M (2008). Mg2+-sensing mechanism of Mg2+ transporter MgtE probed by molecular dynamics study.. Proc Natl Acad Sci U S A.

[27] Scott JW, Hawley SA, Green KA, Anis M, Stewart G (2004). CBS domains form energy-sensing modules whose binding of adenosine ligands is disrupted by disease mutations.. J Clin Invest.

[28] Kemp BE (2004). Bateman domains and adenosine derivatives form a binding contract.. J Clin Invest.

[29] Ignoul S, Eggermont J (2005). CBS domains: structure, function, and pathology in human proteins.. Am J Physiol-Cell Ph.

[30] Sanders PM, Bui AQ, Weterings K, McIntire KN, Hsu YC (1999). Anther developmental defects in Arabidopsis thaliana male-sterile mutants.. Sex Plant Reprod.

[31] Berger F, Hamamura Y, Ingouff M, Higashiyama T (2008). Double fertilization - caught in the act.. Trends Plant Sci.

[32] Kasahara RD, Portereiko MF, Sandaklie-Nikolova L, Rabiger DS, Drews GN (2005). MYB98 is required for pollen tube guidance and synergid cell differentiation in Arabidopsis.. Plant Cell.

[33] Capron A, Gourgues M, Neiva LS, Faure JE, Berger F (2008). Maternal Control of Male-Gamete Delivery in Arabidopsis Involves a Putative GPI-Anchored Protein Encoded by the LORELEI Gene.. Plant Cell.

[34] Zhang YH, Shewry PR, Jones H, Barcelo P, Lazzeri PA (2001). Expression of antisense SnRK1 protein kinase sequence causes abnormal pollen development and male sterility in transgenic barley.. Plant J.

[35] Lu CA, Lin CC, Lee KW, Chen JL, Huang LF (2007). The SnRK1A protein kinase plays a key role in sugar signaling during germination and seedling growth of rice.. Plant Cell.

[36] Thelander M, Olsson T, Ronne H (2004). Snf1-related protein kinase 1 is needed for growth in a normal day-night light cycle.. EMBO J.

[37] McCormick S (1993). Male gametophyte development.. Plant Cell.

[38] Goldberg RB, Beals TP, Sanders PM (1993). Anther development - basic principles and practical applications.. Plant Cell.

[39] Ma H (2005). Molecular genetic analyses of microsporogenesis and microgametogenesis in flowering plants.. Annu Rev Plant Biol.

[40] Halford NG, Paul MJ (2003). Carbon metabolite sensing and signalling.. Plant Biotech Jl.

[41] Weterings K, Russell SD (2004). Experimental analysis of the fertilization process.. Plant Cell.

[42] Pagnussat GC, Yu HJ, Ngo QA, Rajani S, Mayalagu S (2005). Genetic and molecular identification of genes required for female gametophyte development and function in Arabidopsis.. Development.

[43] Punwani JA, Rabiger DS, Drews GN (2007). MYB98 positively regulates a battery of synergid-expressed genes encoding filiform apparatus-localized proteins.. Plant Cell.

[44] Punwani JA, Drews GN (2008). Development and function of the synergid cell.. Sex Plant Reprod.

[45] Jones-Rhoades MW, Borevitz JO, Preuss D (2007). Genome-wide expression profiling of the Arabidopsis female gametophyte identifies families of small, secreted proteins.. PLoS Genet.

[46] Schwab R, Ossowski S, Riester M, Warthmann N, Weigel D (2006). Highly specific gene silencing by artificial microRNAs in Arabidopsis.. Plant Cell.

[47] Yu H, Ito T, Wellmer F, Meyerowitz EM (2004). Repression of AGAMOUS-LIKE 24 is a crucial step in promoting flower development.. Nat Genet.

[48] Clough SJ, Bent AF (1998). Floral dip: a simplified method for Agrobacterium-mediated transformation of Arabidopsis thaliana.. Plant J.

[49] Liu C, Zhou J, Bracha-Drori K, Yalovsky S, Ito T (2007). Specification of Arabidopsis floral meristem identity by repression of flowering time genes.. Development.

[50] Mori T, Kuroiwa H, Higashiyama T, Kuroiwa T (2006). GENERATIVE CELL SPECIFIC 1 is essential for angiosperm fertilization.. Nat Cell Biol.

[51] Yu H, Yang SH, Goh CJ (2000). DOH1, a class 1 knox gene, is required for maintenance of the basic plant architecture and floral transition in orchid.. Plant Cell.

[52] Jakobsen MK, Poulsen LR, Schulz A, Fleurat-Lessard P, Moller A (2005). Pollen development and fertilization in Arabidopsis is dependent on the MALE GAMETOGENESIS IMPAIRED ANTHERS gene encoding a type VP-type ATPase.. Genes Dev.

