# Supplementary figures and images for: AtPV42a and AtPV42b Redundantly Regulate Reproductive Development in Arabidopsis thaliana
Source: PLoS One. 2011 Apr 20;6(4):e19033. doi: 10.1371/journal.pone.0019033 (PMC3080427; doi:10.1371/journal.pone.0019033)

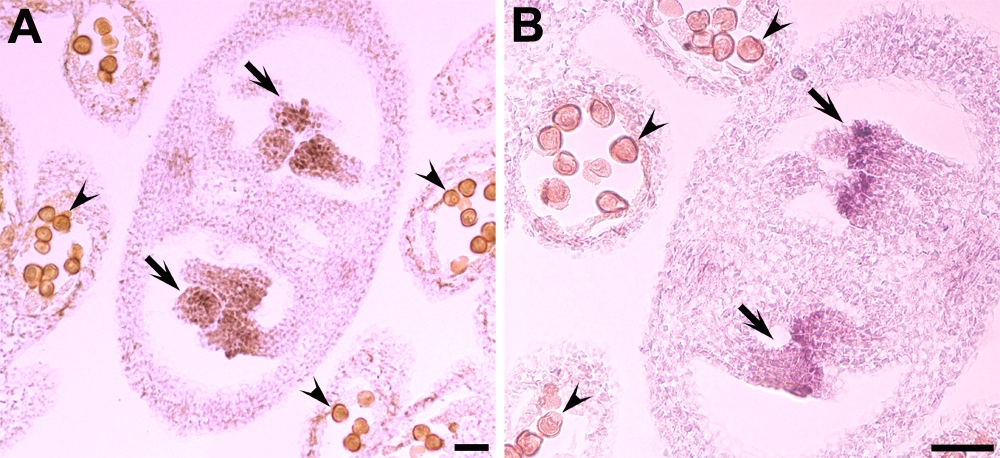

Supplement: Figure S1 — In situ hybridization of AtPV42a and AtPV42b in a stage 11 wild-type flower. A transverse section was hybridized with the antisense AtPV42a (A) or AtPV42b (B) probe. There are hybridization signals inside the gynoecia (arrows), while no signals are detectable in anther cells (arrowheads). Bars, 50 µm. (TIF) [file pone.0019033.s001.tif]

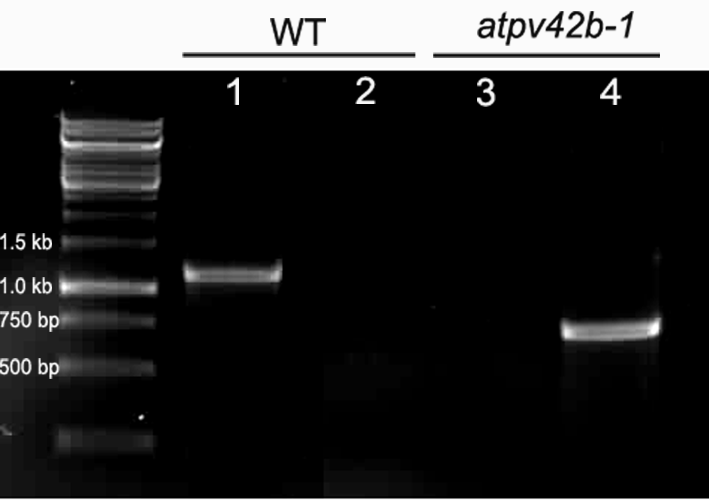

Supplement: Figure S2 — Genotyping of atpv42b-1 mutants using PCR analysis. Lanes 1, 3: PCR products amplified with the left (CS823876_LP) and right (CS823876_RP) primers flanking the AtPV42b genomic region. Lanes 2, 4: PCR products amplified with the T-DNA left border primer (LB2_SAIL) and the right primer for AtPV42b (CS823876_RP). We detected the amplification of a T-DNA fragment (lane 4), but not the AtPV42b genomic region (lane 3) in atpv42b-1, indicating that atpv42b-1 is a homozygous T-DNA insertion mutant. (TIF) [file pone.0019033.s002.tif]

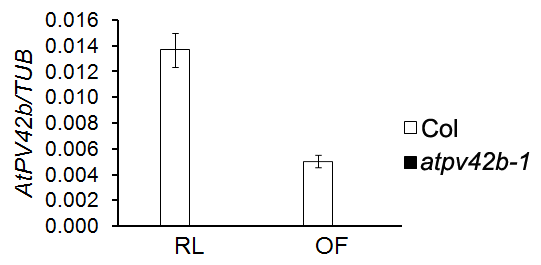

Supplement: Figure S3 — Transcript levels of AtPV42b in rosette leaves (RL) and open flowers (OF) of wild-type and atpv42b-1 plants. AtPV42b expression is undetectable in atpv42b-1 as compared to that in wild-type plants. Transcript levels were determined by real-time PCR using a pair of primers flanking the T-DNA insertion site and are shown relative to TUB2 expression. Values are the mean ± standard deviation from three replicates. (TIF) [file pone.0019033.s003.tif]

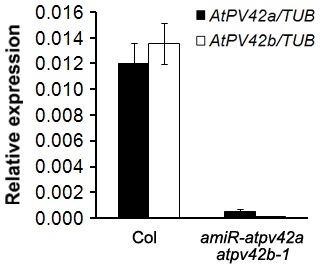

Supplement: Figure S4 — Transcript levels of AtPV42a and AtPV42b in rosette leaves of wild-type and amiR-atpv42a atpv42b-1 plants. The expression of both AtPV42a and AtPV42b is very low in amiR-atpv42a atpv42b-1 as compared to that in wild-type plants. Transcript levels were determined by real-time PCR and are shown relative to TUB2 expression. Values are the mean ± standard deviation from three replicates. (TIF) [file pone.0019033.s004.tif]

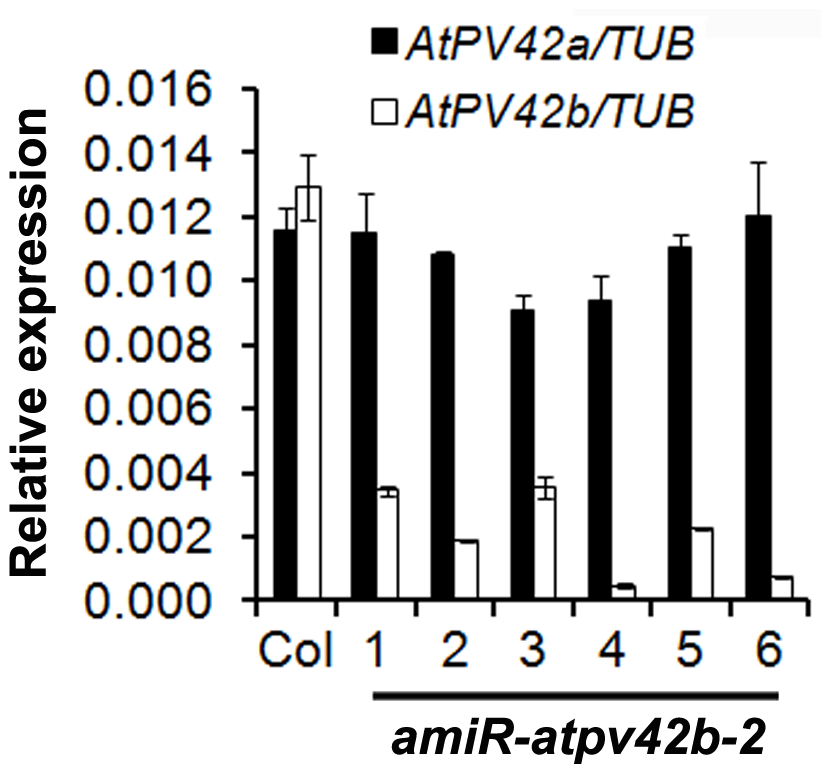

Supplement: Figure S5 — Transcript levels of AtPV42a and AtPV42b in rosette leaves of 6 selected amiR-atpv42b-2 independent transgenic lines at the T1 generation. AtPV42a expression is not significantly affected in these transgenic plants as compared to that in wild-type plants, whereas AtPV42b expression is greatly decreased. Transcript levels were determined by real-time PCR and are shown relative to TUB2 expression. Values are the mean ± standard deviation from three replicates. (TIF) [file pone.0019033.s005.tif]

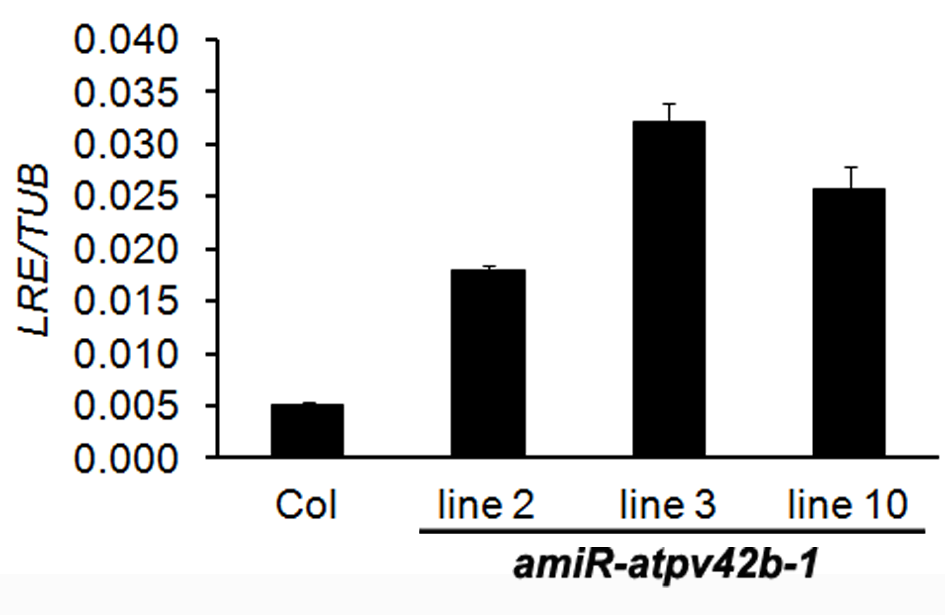

Supplement: Figure S6 — Expression of LRE in open flowers of wild-type and amiR-atpv42b-1 plants. LRE expression is much upregulated in three independent amiR-atpv42b-1 lines than in wild-type plants. Transcript levels were determined by real-time PCR and are shown relative to TUB2 expression. Values are the mean ± standard deviation from three replicates. (TIF) [file pone.0019033.s006.tif]
