# Supplementary material for: AtPV42a and AtPV42b Redundantly Regulate Reproductive Development in Arabidopsis thaliana
Source: PLoS One. 2011 Apr 20;6(4):e19033. doi: 10.1371/journal.pone.0019033 (PMC3080427; doi:10.1371/journal.pone.0019033)
Supplement: Table S1 — Phenotypic analysis of amiR-atpv42b-1 pollen grains. (DOC) [file pone.0019033.s007.doc]

**Table S1.** Phenotypic analysis of *amiR-atpv42b-1* pollen grains.

|  | Col | *amiR-atpv42b-1* (line 10) |
| --- | --- | --- |
| Normal | 795 (95.2%) | 350 (30.8%) |
| Sterile | 40 (4.8%) | 785 (69.2%) |

For each genotype, pollen grains from at least 10 flowers at the anthesis stage were examined.
