# Supplementary material for: AtPV42a and AtPV42b Redundantly Regulate Reproductive Development in Arabidopsis thaliana
Source: PLoS One. 2011 Apr 20;6(4):e19033. doi: 10.1371/journal.pone.0019033 (PMC3080427; doi:10.1371/journal.pone.0019033)
Supplement: Table S2 — Primers used in this study. (DOC) [file pone.0019033.s008.doc]

**Table S2.** Primers used in this study.

| **Primer Name** | **Sequence (5’-3’)** | **Application** |
| --- | --- | --- |
| CS823876_LP | TACGCAATGCTGAGTCAGATG | Genotyping  of *atpv42b-1* |
| CS823876_RP | GTATGTCATGCTTCTTTGCGC |
| LB2_SAIL | GCTTCCTATTATATCTTCCCAAATTACCAATACA |
| AtPV42amiR-s | gaTGAAAACGTACCTATCACTTCtctctcttttgtattcc | Generation of *amiR-atpv42a* |
| AtPV42amiR-a | gaGAAGTGATAGGTACGTTTTCAtcaaagagaatcaatga |
| AtPV42amiR-*s | gaGACGTGATAGGTAGGTTTTCTtcacaggtcgtgatatg |
| AtPV42amiR-*a | gaAGAAAACCTACCTATCACGTCtctacatatatattcct |
| AtPV42bmiR-s | gaTGAATAGTCATAGTGTTCAGGtctctcttttgtattcc | Generation of *amiR-atpv42b-1* |
| AtPV42bmiR-a | gaCCTGAACACTATGACTATTCAtcaaagagaatcaatga |
| AtPV42bmiR-*s | gaCCCGAACACTATGTCTATTCTtcacaggtcgtgatatg |
| AtPV42bmiR-*a | gaAGAATAGACATAGTGTTCGGGtctacatatatattcct |
| AtPV42bmiR-1 | gaTTACTGTCCAATGGGACCGATtctctcttttgtattcc | Generation of *amiR-atpv42b-2* |
| AtPV42bmiR-2 | gaATCGGTCCCATTGGACAGTAAtcaaagagaatcaatga |
| AtPV42bmiR-3 | gaATAGGTCCCATTGCACAGTATtcacaggtcgtgatatg |
| AtPV42bmiR-4 | gaATACTGTGCAATGGGACCTATtctacatatatattcct |
| TUB2-RTF | ATCCGTGAAGAGTACCCAGAT | Real-time PCR |
| TUB2-RTR | AAGAACCATGCACTCATCAGC |
| AtPV42a-RTF | GGGATTCTCACGATGCTTGAC |
| AtPV42a-RTR | TGTCCAGAGACTGAGTCCTTCG |
| AtPV42b-RTF | GAGAGGATCATAAACAGCTCGTC |
| AtPV42b-RTR | CTCGACGAACTCTAGTGCATTT |
| LRE-RTF | TTCTTTCTGATGGCACTGTTG |
| LRE-RTR | AACCGAGGTTTGTGATTCAA |
| AtPV42a-IS-F | CTTGCGTTTCCTTAAAGACCA | Generation of probes for  in situ hybridization |
| AtPV42a-IS-R | ATGATATCAGTGAGGGAGACGAC |
| AtPV42b-IS-F | TTCTTCGACCAATCCTCTCAA |
| AtPV42b-IS-R | GACCACAGCGATGATGTCAG |
